# Real-time alerting system for COVID-19 and other stress events using wearable data

**DOI:** 10.1038/s41591-021-01593-2

**Published:** 2021-11-29

**Authors:** Arash Alavi, Gireesh K. Bogu, Meng Wang, Ekanath Srihari Rangan, Andrew W. Brooks, Qiwen Wang, Emily Higgs, Alessandra Celli, Tejaswini Mishra, Ahmed A. Metwally, Kexin Cha, Peter Knowles, Amir A. Alavi, Rajat Bhasin, Shrinivas Panchamukhi, Diego Celis, Tagore Aditya, Alexander Honkala, Benjamin Rolnik, Erika Hunting, Orit Dagan-Rosenfeld, Arshdeep Chauhan, Jessi W. Li, Caroline Bejikian, Vandhana Krishnan, Lettie McGuire, Xiao Li, Amir Bahmani, Michael P. Snyder

**Affiliations:** 1 Department of Genetics, Stanford University School of Medicine, Stanford, CA USA; 2 Department of Computer Science, Stanford University, Stanford, CA USA; 3 Department of Biochemistry, Case Western University, Cleveland, OH USA; 4 Center for RNA Science and Therapeutics, Case Western University, Cleveland, OH USA; 5 Department of Computer and Data Sciences, Case Western University, Cleveland, OH USA

**Keywords:** Predictive markers, Infectious diseases

## Abstract

Early detection of infectious diseases is crucial for reducing transmission and facilitating early intervention. In this study, we built a real-time smartwatch-based alerting system that detects aberrant physiological and activity signals (heart rates and steps) associated with the onset of early infection and implemented this system in a prospective study. In a cohort of 3,318 participants, of whom 84 were infected with severe acute respiratory syndrome coronavirus 2 (SARS-CoV-2), this system generated alerts for pre-symptomatic and asymptomatic SARS-CoV-2 infection in 67 (80%) of the infected individuals. Pre-symptomatic signals were observed at a median of 3 days before symptom onset. Examination of detailed survey responses provided by the participants revealed that other respiratory infections as well as events not associated with infection, such as stress, alcohol consumption and travel, could also trigger alerts, albeit at a much lower mean frequency (1.15 alert days per person compared to 3.42 alert days per person for coronavirus disease 2019 cases). Thus, analysis of smartwatch signals by an online detection algorithm provides advance warning of SARS-CoV-2 infection in a high percentage of cases. This study shows that a real-time alerting system can be used for early detection of infection and other stressors and employed on an open-source platform that is scalable to millions of users.

## Abstract (web summary)

In a prospective study, a smartwatch-based alerting system was able to detect pre-symptomatic and asymptomatic SARS-CoV-2 infection in a high percentage of cases.

## Main

Early detection of infectious diseases helps prevent transmission and enable early intervention. Traditionally, detection has been limited to symptom onset when physiological disturbances often warrant medical attention and disease transmission might already have occurred. For respiratory viral infections, symptom onset is typically several days to over 1 week after infection, whereas asymptomatic infections are not likely to be detected at all^1–3^. When a symptom onset does occur, it is usually followed up by either an oral or skin temperature measurement or more definitively diagnosed using a biochemical test, such as antigen detection or polymerase chain reaction (PCR)^4,5^.

Wearable devices such as smartwatches have the potential to monitor individuals continuously in real time and thus provide early detection of respiratory illnesses and other infections^6–11^. These devices can collect different types of physiological data, such as heart rate, step counts, sleep and temperature. Recent studies have shown that wearables can be used to identify early signs of infectious diseases such as Lyme disease^6^ or respiratory viral infections, including coronavirus disease 2019 (COVID-19)^7–10^, and might even permit pre-symptomatic detection^6,7^. These respiratory viral infection studies have focused primarily on detection at symptom onset and, in the case of pre-symptomatic detection, were performed retrospectively. So far, the ability to prospectively detect respiratory viral infections and other stress events has not been examined, nor has a system been developed for performing this at scale. An early detection approach using a monitoring and alerting system can enable early self-isolation, treatment and effective allocation of healthcare resources and provide an invaluable tool for potentially containing pandemics.

In this study, we created, to our knowledge, the first large-scale, real-time monitoring and alerting system for detecting abnormal physiological events, including COVID-19 infection onset, using agnostic algorithms across different types of smartwatches. We designed a novel algorithm capable of detecting outlier measurements associated with physiological stresses in real time, including COVID-19 and other respiratory illnesses, and generating alerts for the device wearer. For pre-symptomatic cases, we show that the system identifies approximately 80% of COVID-19 illnesses at or before the onset of symptoms. It also identifies asymptomatic cases and signals resulting from other stressors, such as vaccination. Associations between symptoms and activities with alerting signals were also investigated.

## Results

### Study overview

**Fig. 1:**
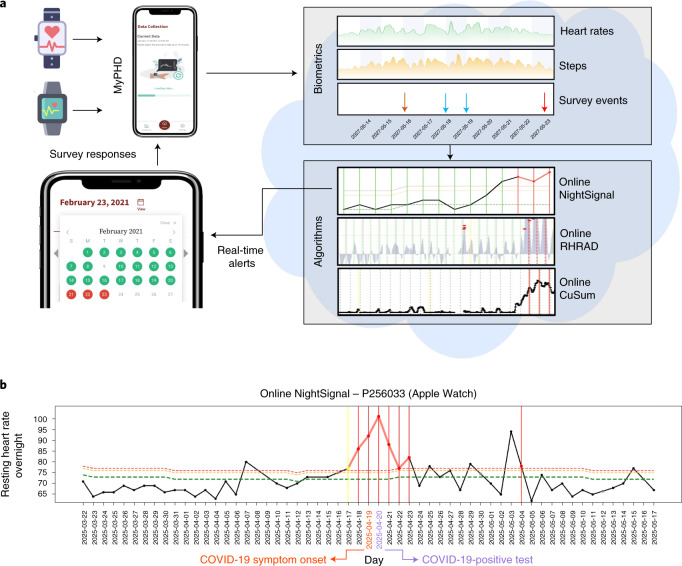
Study overview. **a**, Participants with a Fitbit and/or Apple Watch were asked to share their wearable and survey data using the study mobile app MyPHD. The app securely transfers the de-identified data (heart rates, steps and survey events) to the back-end for real-time analysis. On the back-end, three online infection detection algorithms were deployed, and the results from one of the algorithms (online NightSignal) were returned to the participants using the app: red alerts indicate abnormal changes in overnight RHR; green alerts indicate normal overnight RHR. **b**, A real-world example of real-time pre-symptomatic detection of COVID-19 using the online NightSignal algorithm for a participant using an Apple Watch. Alerts were triggered 2 d before the symptom onset date and continued until 3 d after the diagnosis date.

We constructed a highly secure, real-time alerting system for detecting abnormal periods of stress, such as viral infections, using wearable devices (Fig. 1a) and conducted a test study approved by the Stanford University institutional review board (IRB) (protocol no. 57022) and Data Risk Assessment (DRA no. 665). The system involves participant enrollment through a secure REDCap^12^ e-consent system using the study app, called MyPHD^13,14^. After connecting the smartwatch through the app, wearable data (heart rate, step count and sleep analysis) and health information (for example, surveys of illness, symptom, medication and vaccination) were collected and securely transferred in real time to the cloud for further analysis. Three online infection detection algorithms (NightSignal, RHRAD and CuSum) were hosted on the cloud, and the results of one of them, NightSignal, were made available to the participants in the form of real-time alerts (that is, red or green alert per day; an example of a signal associated with the alerts in an individual who was positive for COVID-19 is shown in Fig. 1b). Participants were expected to annotate the alerts using several surveys (COVID-19 test, activities and symptoms). Medical recommendations (for example, self-isolation and getting tested) were not allowed under our IRB protocol.

**Extended Data Fig. 1:**
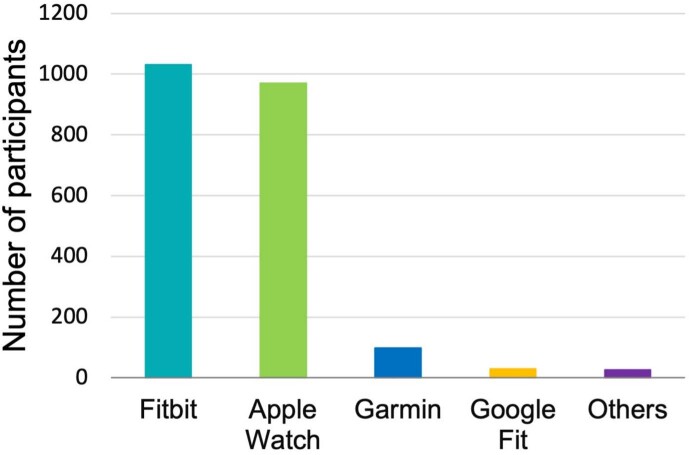
Wearable devices distribution. Of the 2,155 participants who had a smartwatch: 1,031 wore Fitbits, 970 wore Apple Watches, 98 wore Garmin, and 56 had other devices. Note that we consider the device with the most amount of data as the main device in case of having more than one device.

**Extended Data Fig. 2:**
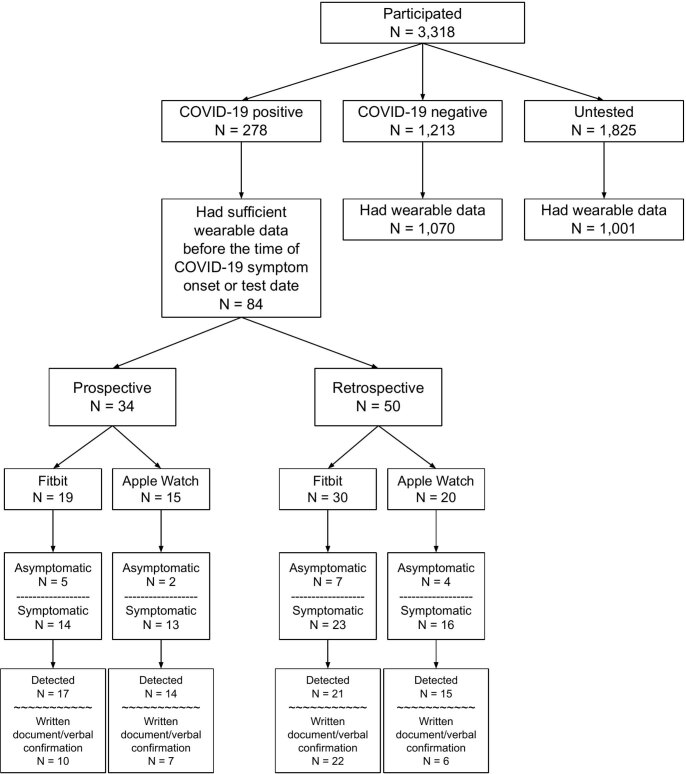
Flow chart of participants. A flow chart over the participants in the study including the number of participants with sufficient wearable data in each category — in COVID-19 positive cases, if there are more than two days missing in a week before symptom onset (for the symptomatic cases) or more than a week missing data in 21 days before the diagnosis date (for the asymptomatic cases), we consider the case as insufficient data — and number of participants in each sub-category of COVID-19 positive cases: prospective/retrospective, Fitbit/Apple Watch, and symptomatic/asymptomatic. As shown in the figure, 66 participants were symptomatic and 18 were asymptomatic. 45 participants (5 asymptomatic) confirmed diagnosis of COVID-19 via written documentation or verbal confirmation of their test result.

We enrolled a total of 3,318 participants between 27 November 2020 and 20 July 2021, of whom 2,155 had wearable data. Of these, 1,031, 970 and 98 wore Fitbit, Apple Watch or Garmin watches, respectively, and the remaining wore other devices (Extended Data Fig. 1). During the study, 2,117 participants received daily real-time alerts for physiological changes, and 2,122 participants filled in at least one survey. Of all participants, 278 individuals reported COVID-19-positive test results (84 were confirmed by written documentation (62 individuals) or verbal confirmation of their test result (22 individuals)). Of those participants reporting COVID-19-positive tests, 84 had sufficient wearable data around the time of COVID-19 infection for alert signaling (49 Fitbit cases and 35 Apple Watch cases) (Extended Data Fig. 2). Fifty of these cases were retrospective, whereby individuals tested positive before enrollment; in 34 cases, the individuals tested positive after enrollment.

We also analyzed wearable data for three other categories of participants: (1) individuals who tested negative for COVID-19: 1,213 participants who reported a COVID-19-negative test and never had a positive test (individuals who tested negative for COVID-19 reported their diagnosis date via the COVID-19 survey in the study app); (2) untested individuals: 1,825 participants without any COVID-19 test report; and (3) vaccinated individuals: 189 participants who received the COVID-19 vaccine (Moderna or Pfizer-BioNTech), of whom 182 were fully vaccinated (that is, both doses) (Supplementary Table 1). COVID-19-positive, COVID-19-negative and untested groups are distinct datasets. Vaccinated individuals could be COVID-19-positive, COVID-19-negative or untested.

Participants who received an alert were expected to provide a description of their diagnosis, symptoms and activities during that period. The above diagnoses included COVID-19, adenovirus and influenza. The symptoms included cough, fever and headache as well as severity (1—mild to 5—very severe). The activities included intense exercise, alcohol consumption, travel, stress and other lifestyle factors that could alter physiological signals.

### COVID-19 triggers real-time alerts

**Extended Data Fig. 3:**
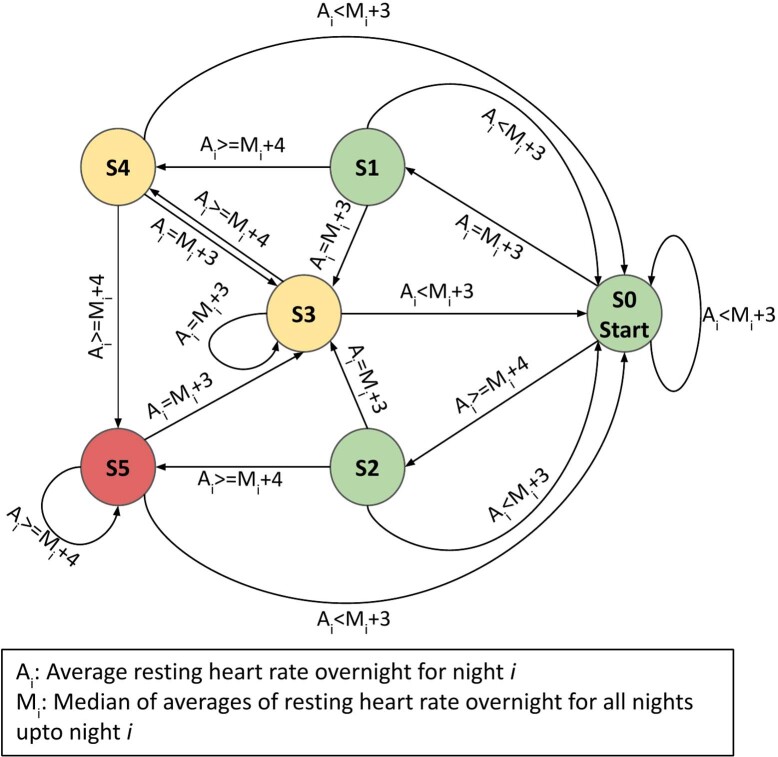
Deterministic Finite State Machine in NightSignal algorithm. The state machine consists of six states, each labeled with an alert color and three symbols for transition between states based on the current average RHR overnight and the deviation level from the baseline (streaming median of averages of RHR overnight). For example, a red alert gets triggered if for two consecutive nights, the average RHR overnight is at least four bpm above the calculated baseline.

We used three independent real-time alerting algorithms capable of detecting and tracking physiological changes due to infections such as COVID-19. Two algorithms, online RHRAD and CuSum, extended from our previous work^7^, detect abnormal deviations from the baseline in resting heart rates (RHRs) using distinct approaches (Methods). These two methods were applied on the dense Fitbit data and have the potential to report an alarm at hourly resolution. Although they can detect anomalies at high resolution, these algorithms are computationally intensive. To be less computationally intensive and potentially scalable to millions of users with various smartwatches, as well as achieving higher sensitivity while keeping the false-positive (FP) rate minimal, we developed a novel ‘lightweight’ algorithm (NightSignal) that uses a deterministic finite state machine (FSM)^15^ based on overnight RHR (Methods and Extended Data Fig. 3). For each individual, we use the streaming median of average overnight RHR as the baseline and raise real-time daily alerts as deviations from baseline as defined by the alerting state machine (Methods). Deviations in successive nights (31 h) trigger an alert. This algorithm runs on both the Fitbit and Apple Watch, and its features are presented in Extended Data Fig. 3. The NightSignal method has high sensitivity (80%) compared to CuSum (72%) and RHRAD (69%) but produces the same rate of false non-COVID-19 signals (Supplementary Table 2).

### Real-time pre-symptomatic and asymptomatic detection

**Fig. 2:**
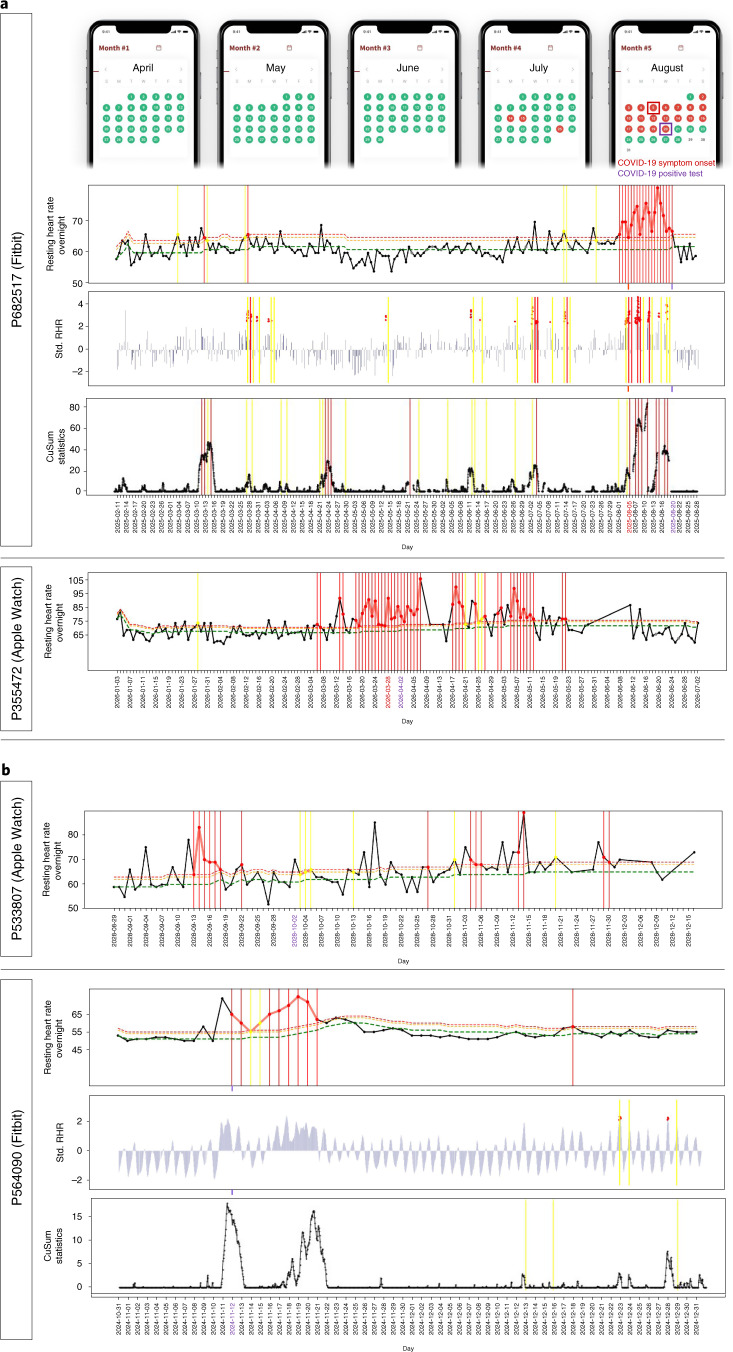
Examples of COVID-19 real-time pre-symptomatic and asymptomatic detection. **a**, Pe-symptomatic detection for participants who tested positive for SARS-CoV-2 using a Fitbit (top) and an Apple Watch (bottom). For the participant using a Fitbit, the panels show data derived using the online NightSignal, online RHRAD and online CuSum algorithms. Alerts generated by the NightSignal algorithm initiated 3 d before symptom onset and persisted for the next 15 d. For the participant using an Apple Watch, the panel shows data derived using the online NightSignal algorithm. Alerts appeared 10 d before symptom onset and continued until 10 d after that. **b**, Asymptomatic detection for participants who tested positive for SARS-CoV-2 using an Apple Watch (top) or a Fitbit (bottom). For the participant using an Apple Watch, the panel shows data derived using the online NightSignal algorithm. For the participant using a Fitbit, the panels show data derived using the online NightSignal, online RHRAD and online CuSum algorithms.

We first examined the capability of the real-time alerting system in the detection of COVID-19 at an early, pre-symptomatic stage as well as in asymptomatic cases. Figure 2a shows two examples of prospective detection confirmed by COVID-19-positive tests: a Fitbit case (top) and an Apple Watch case (bottom) in which we detected elevated NightSignal alerts starting 3 d and 10 d before symptom onset, respectively. The screenshots of the MyPHD app at the top of Fig. 2a show the real-time alerts that the corresponding participant received every day. Alerts are visualized via a calendar, and participants annotate the alerts using different surveys in the app, including activities, symptoms, diagnosis, medications and vaccination surveys. For the Fitbit example, all three algorithms raised alerts before symptom onset.

We also found 18 cases in which individuals reported testing positive but were asymptomatic. Alert signals were found to be associated with positive diagnostic tests in 14 of these cases. Two examples of asymptomatic participants who tested positive for COVID-19 are shown in Fig. 2b, for whom the alerts triggered 19 d before and on the COVID-19 diagnosis date, respectively. For the Fitbit example, only the NightSignal algorithm raises consecutive COVID-19-related alerts because the other two algorithms required more baseline data.

**Fig. 3:**
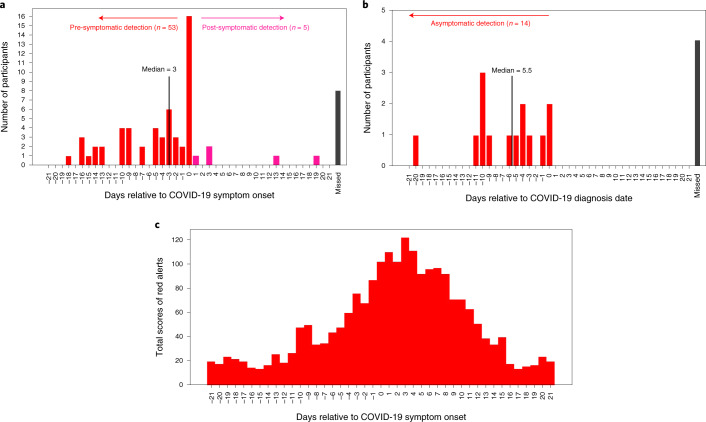
Association of red alerts with COVID-19 symptoms and diagnosis. **a**, Association of the initiation of red alerts in the NightSignal algorithm with COVID-19 symptom onset in 66 participants who tested positive for SARS-CoV-2 with symptoms using a Fitbit or an Apple Watch, with respect to a window of time centered around symptom onset (21 d before to 21 d after symptom onset). The NightSignal algorithm achieved pre-symptomatic detection in 53 participants and post-symptomatic detection in five participants; eight participants did not receive any red alert associated with their COVID-19 symptoms during the detection window. **b**, Association of the initiation of red alerts in the NightSignal algorithm with a SARS-CoV-2-positive test in asymptomatic participants using a Fitbit or an Apple Watch. The plot shows 21 d before and 21 d after the COVID-19 diagnosis date. The NightSignal algorithm achieved asymptomatic detection in 14 participants; four participants did not receive any red alert associated with their COVID-19 diagnosis during the detection window. **c**, The distribution of scores of red alerts with respect to a window of 21 d before and 21 d after the COVID-19 symptom onset date in participants who tested positive for SARS-CoV-2. Red bars indicate the cumulative scores of red alerts, as described in the Methods.

A summary of alert signals for all pre-symptomatic and asymptomatic cases is shown in Fig. 3. To increase detection power, we included the results from 50 retrospective cases where individuals tested positive before study enrollment, as well as 34 cases where individuals tested positive after enrollment (results were similar among the two groups (Extended Data Fig. 2). Of these, 49 were Fitbit users and 35 were Apple Watch users. Owing to the unknown virus exposure time, we cannot define the precise infectious period for participants who tested positive for COVID-19. Therefore, for the participants who tested positive for COVID-19, we defined true positives (TPs) as the number of cases that received red alerts from the algorithm before or at COVID-19 symptom onset (for pre-symptomatic cases) or diagnosis date (for asymptomatic cases) with respect to the infection detection window (that is, 21 d before the symptom onset for symptomatic cases or diagnosis date for asymptomatic cases). We defined false negatives (FNs) as the number of participants who did not receive any alert within the same infection detection window. For the participants who tested negative for COVID-19 as well as untested participants, we defined true negatives (TNs) as the number of green alerts that were correctly sent to these participants during non-COVID-19 periods (that is, 21 d before a negative test result for individuals who tested negative for COVID-19, the entire time frame for untested participants and days before the infection detection window for COVID-19-positive cases). We also defined FPs as the number of red alerts that were incorrectly sent to these participants during the above-mentioned non-COVID-19 periods. Of the 84 participants who tested positive for COVID-19 (66 symptomatic and 18 asymptomatic), 67 received NightSignal alerts at or before symptom onset (for pre-symptomatic cases) or diagnosis date (for asymptomatic cases) for a sensitivity (TP rate) of 80% (Fig. 3a,b). The number was similar for Fitbit (77%) and Apple Watch (83%). For pre-symptomatic cases, an additional five cases received alerts within 21 d after symptom onset, and eight cases did not yield any NightSignal alerts during the infection period (−21 d to +21 d around the onset of symptoms). We note that the lack of sufficient wearable data might have been a reason for some of these missed cases.

Unlike the NightSignal algorithm, which does not require high-resolution data and thus functions on different devices with different resolutions (for example, Fitbit and Apple Watch), the online RHRAD and CuSum algorithms function only on Fitbit data. For the case of Fitbit, which had hourly data, we were able to analyze the sensitivity of the online RHRAD and CuSum algorithms; these were found to be 29/42 (69%) and 29/40 (72%), respectively. To measure the performance of the algorithms on participants who tested negative for COVID-19 as well as untested participants, the specificity (TN rate) of the algorithms defined as TN/(TN + FP) is as follows: NightSignal 87,124/(87,124 + 12,186) = 87.7%; online RHRAD 57,108/(57,108 + 7,971) = 87.7%; and online CuSum 35,451/(35,451 + 6,889) = 83.7%. In addition to these three algorithms, we also retrospectively benchmarked the NightSignal algorithm against the Isolation Forest anomaly detection algorithm, which is commonly used to detect anomalies in the data^16^. A detailed summary of performance comparison is shown in Supplementary Table 2. The Night Signal algorithm performed favorably compared to the Isolation Forest method.

To determine the FP (non-COVID-19) rate, in addition to the above population-alert-based FP and TN, we calculated the specificity in an individual-based analysis where we report the average of TN rates across participants. Similarly, we also report the FP rate for the COVID-19-positive population for the period before their COVID-19 detection window. As described below, these values are with respect to COVID-19 detection and other biologically relevant events that could trigger these alerts. Supplementary Table 3 shows a comparison between the mean alerts received by an individual in the three categories mentioned above, in a 21-d window. An individual who tested positive for COVID-19 receives 3.42 alerts on average during the infection detection window, whereas this number is 1.30 for individuals who tested negative for COVID-19 and 1.09 for the remainder. Thus, COVID-19-positives have a higher mean alert rate.

To examine the alerting period relative to symptom onset, we calculated the scores of red alerts based on a cumulative scoring system (Methods) and plotted the distribution with respect to the period of time centered around symptom onset (Fig. 3c and Methods). Across the 66 symptomatic participants, we observed that the maximum number of clustered alerts occur in a window of −4 d to +11 d around symptom onset.

### Symptoms and activities raise RHR signals

**Fig. 4:**
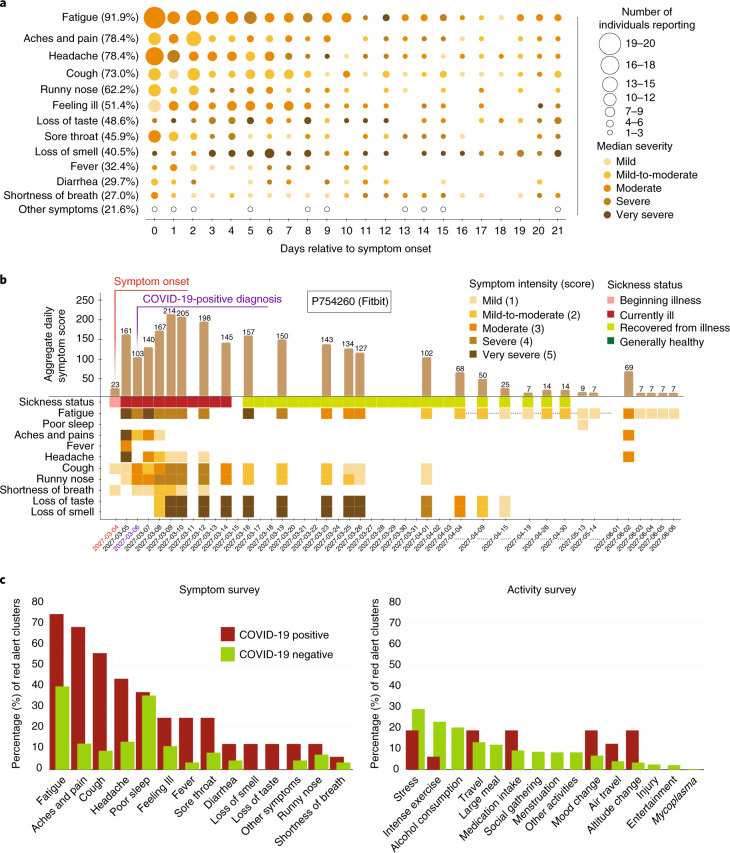
Association of clustered red alerts with symptom progression. **a**, Bubble plot showing day-by-day frequency counts of individuals reporting symptoms during the second half of the infection detection window (from symptom onset to 21 d later). Bubble size and shading are indicative of the relative magnitude of the frequency count and the median severity, respectively. The percentage in the brackets alongside each symptom indicates the total number of individuals reporting that symptom over all 21 d as a fraction of the total number of symptomatic participants who tested positive for SARS-CoV-2. **b**, Illustrative example tracing the symptoms of a participant who tested positive for SARS-CoV-2 from symptom onset to 21 d later and continuing thereafter intermittently for an additional 2 months. For each day, an aggregate symptom score was computed as the sum of the relative severity of the symptoms, each weighted by its specificity to individuals who tested positive for SARS-CoV-2. The scale for computing the symptom score was based on individual symptom intensities, each ranging from mild (score = 1) to very severe (score = 5) as reported by the participant. The aggregated score shown in this bar plot is a measure of the overall severity. **c**, The bar plots show the percentages of red alert periods (from NightSignal algorithm) associated with each symptom (left) or activity (right) as annotated by participants who tested positive for COVID-19 as well as by participants who tested negative for COVID-19. Source data

A wide variety of symptoms are associated with COVID-19 (refs. ^17,18^). To examine illness progression, we aggregated symptoms by both severity and number of individuals reporting across 21 d relative to symptom onset (Fig. 4a). Consistent with the literature^19,20^, most symptoms were evident in the first 7–8 d of illness (fatigue, headache and feeling ill), although fatigue often continued well after symptom onset. Notably, fatigue was the most commonly reported symptom, whereas loss of smell or taste seem to be the highest in terms of severity. An example of an individual with many symptoms, some which persisted for 3 or more weeks, is shown in Fig. 4b.

We examined the red alert clusters (two or more consecutive red alert days) annotated by participants who tested positive for COVID-19 vis-a-vis those annotated by participants who tested negative for COVID-19. The distribution of symptoms and activities across the clusters was quite different depending on COVID-19 diagnosis (Fig. 4c). Symptoms such as fatigue and poor sleep were generally present for both COVID-19-positive and COVID-negative cases, but aches and pains, headaches, cough and feeling ill were less frequent in the COVID-19-negative cases. Stress, intense exercise and alcohol consumption were activities most commonly associated with red alerts in individuals with COVID-19-negative diagnoses. Furthermore, the mean duration of such annotated red alert clusters was much higher (6.2 d) for individuals who tested positive for COVID-19 than individuals who tested negative for COVID-19 negatives (3.5 d) and untested individuals (3.7 d).

**Fig. 5:**
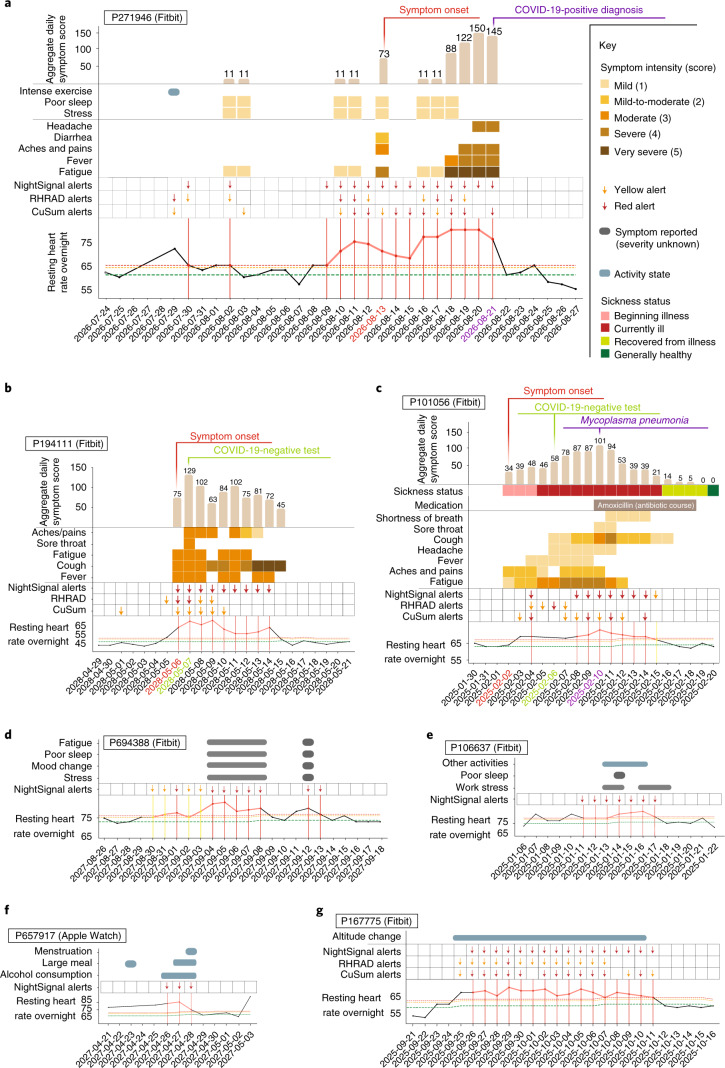
Association between RHR elevation with symptoms and activities. **a**, Example of a SARS-CoV-2-positive case. Alerts began before symptom onset and continued until the diagnosis date. Elevated RHR was associated with severe fatigue, fever and headache. **b**, Example of a SARS-CoV-2-negative case. Even though alerts were present throughout the symptom period, the magnitude of RHR elevation is noticeably lower compared to the SARS-CoV-2-positive case in **a**. **c**, Example of alerts associated with *M. pneumonia* infection. On the 4th day after symptom onset, the participant received a negative test for SARS-CoV-2. *M. pneumonia* was detected on the 8th day after symptom onset, and both symptoms and alerts were receded on the 14th day after symptom onset (5 d after antibiotic therapy). **d**, **e**, Examples illustrating associations of stress, poor sleep and mood change with the occurrence of alerts in the individuals who tested negative for SARS-CoV-2. **f**, Example illustrating the association between repeated alcohol consumption and alerts in an individual who tested negative for SARS-CoV-2. **g**, Example illustrating extended altitude change and alerts in an individual who tested negative for SARS-CoV-2.

Examples of the alerts and symptoms signals are shown longitudinally from a COVID-19-positive case (Fig. 5a), a COVID-19-negative case (Fig. 5b), a diagnosed *Mycoplasma pneumoniae* infection (Fig. 5c), individuals annotating stress or work stress (Fig. 5d,e), repeated alcohol consumption (Fig. 5f) and extended altitude change (Fig. 5g). These results indicate that other events can be attributed to red alerts when surveys are regularly reported. In many of these cases (for example, alcohol, altitude or other stresses), these events are easy for the users to contextualize. It is also important to note that the RHR elevation during the red alert cluster is noticeably higher for the COVID-19-positive case than for the COVID-19-negative and *Mycoplasma* cases. More examples of different categories (COVID-19-positive, COVID-19-negative and untested) are shown in Extended Data Fig. 6. Finally, as reported previously, we note that many non-COVID-19 alerts are evident over the winter holidays—more than other times of the year (a 1.4-fold increase in red alerts; Extended Data Fig. 8). In this study, this increase was evident during the pandemic, whereas this was noted before the pandemic in our previous study^7^, indicating that these events occur independent of the pandemic. It is possible that these holiday-associated events are due to increased stress, alcohol consumption and/or travel.

### COVID-19 vaccination often yields real-time alerts

There is considerable interest in understanding how response to vaccination compares to actual infections; wearable data provide an opportunity to investigate physiological responses to vaccination in the real world. On 11 December 2020 and 18 December 2020, the US Food and Drug Administration issued the first emergency use authorization for the Pfizer-BioNTech and Moderna COVID-19 vaccines, respectively^21,22^. These randomized vaccine clinical trials showed 94–95% efficacy in preventing COVID-19 illness. Overall, localized side effects (for example, aches and rash) after vaccination have been shown to be mild; however, moderate-to-severe systemic side effects, such as fatigue and headache, were reported by some participants^22^ and provide an opportunity to examine the ability of our algorithms to detect COVID-19 vaccination-related events.

**Fig. 6:**
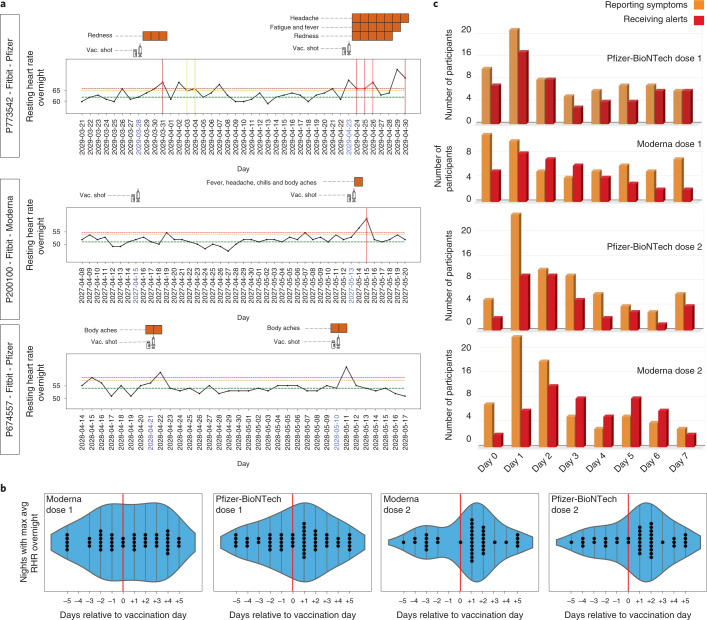
Association of red alerts with COVID-19 vaccination. **a**, Examples of the association of COVID-19 vaccination with alerts from the online NightSignal algorithm. **b**, Effects of COVID-19 vaccination on average RHR overnight in the cases of (from left to right) the first dose of the Moderna vaccine, the first dose of the Pfizer-BioNTech vaccine, the second dose of the Moderna vaccine and the second dose of the Pfizer-BioNTech vaccine. **c**, Distribution of symptoms reported and alerts received for 1 week after the first and second doses of the Pfizer-BioNTech and Moderna vaccines.

In early January 2021, we added COVID-19 vaccination surveys to the study app, MyPHD. As expected, alerts were triggered one to several days after vaccination in many participants. Three examples of the possible effects of vaccination detected by the NightSignal algorithm and the related symptoms are shown in Fig. 6a. As shown in these longitudinal examples, vaccination can trigger the alerts after both doses or after only one dose; however, in some cases, RHR overnight might increase only for a short period (for example, one night), and, hence, no alert is raised. To determine the effect of vaccination on RHR overnight, we analyzed the average RHR overnight for 5 d before and after the vaccination date. Interestingly, we observed that, for the first dose, the maximum RHR overnight occurred the night of the vaccination in the case of the Pfizer-BioNTech vaccine (an increase was not evident for Moderna); for the second dose, it occurred the first or second night after vaccination for the Moderna and Pfizer-BioNTech vaccines, respectively (46% in Moderna and 54% in Pfizer-BioNTech; Fig. 6b).

The symptoms reported for 1 week after vaccination were recorded from the surveys. After the first dose, the most frequently occurring symptoms with either vaccine are fatigue, poor sleep, aches and pain. After the second dose, fatigue, headache, aches and pain top the list. There are also some striking differences between Pfizer-BioNTech and Moderna. In the case of Pfizer-BioNTech, fever is reported after either dose, whereas, for Moderna, fever was reported less frequently after the first dose, but almost 60% of the participants reported a fever after the second dose. The distribution of symptoms reported and alerts received for 1 week after the first and second dose of COVID-19 vaccines is shown in Fig. 6c. Symptoms and alerts show similar trends, with the maximum number of alerts occurring in the first or second night after the vaccination. Overall, these results show the presence of symptoms associated with vaccination, particularly the second dose, and that the effects of vaccination are readily detected using a smartwatch.

## Discussion

Here we introduce the first prospective, real-time physiological stress detection and alerting system that can detect early-onset illness using a smartwatch. It detects COVID-19 at or before symptoms in approximately 80% of the symptomatic cases and even identifies asymptomatic cases; this is the first time, to our knowledge, that asymptomatic detection has been shown for COVID-19, although it has been reported for other infections^6^. The actual number of asymptomatic cases is difficult to judge because most such cases are likely not tested with RT–PCR; nonetheless, we found that 14 of 18 asymptomatic cases had alerts near the test date (within 21 d before the diagnosis date). Detection results were similar for Fitbit and Apple Watch. In this study, medical recommendations were not provided to participants, although implementation in future studies might allow this. Alerts were generated sufficiently early—a median of 3 d before symptom onset for COVID-19 cases—to enable effective early self-isolation and testing.

Many of the alert-generating events detected in this study were not associated with COVID-19. Most of the annotated alerts can be attributed to other events, such as poor sleep, stress, alcohol consumption, intense exercise, travel or other activities. In many of these cases, the alerting events would be easy to self-contextualize (intense exercise, alcohol consumption and travel), and the participants would be unlikely to take action. In other cases, such as COVID-19-negative diagnoses with symptomatic illness, follow-up testing would be expected to be valuable.

From a survey of all participants who received alerts, 73% found that the frequency of red alerts was acceptable, and they usually could link the alerts to abnormal events (Supplementary Table 4). They did not experience alarm fatigue for the relative short duration of this study (less than 1 year). In the future, we plan to allow users to set the sensitivity threshold. Thus, for example, healthcare workers might choose a higher frequency for early detection, whereas others might choose a lower frequency.

The large number of unannotated alerts might be due to (1) failure to annotate an alert, (2) asymptomatic infections or (3) other stressors. Because stress can trigger increases in RHR, which is a major feature of our detection algorithms, this approach can potentially be used to monitor mental health as well as physical health. In addition, due to the lightweight feature of the proposed algorithm, the analysis of data from the smartwatch can be performed directly on the user’s phone, thereby substantially reducing the cost of the back-end systems’ operation and maintenance.

It is unclear why COVID-19 is not detected in all cases. Some of these individuals have an unstable baseline (Methods), and, for others, the abnormal signals deviating from the baseline are not large enough to generate a signal. The inclusion of higher-resolution data and/or other data types, such as heart rate variability, respiration rate, skin temperature, SpO_2_ changes, galvanic stress response or other physiological features, are expected to improve detection performance for both the number of events and the earliest detection time. Such data will likely help distinguish the COVID-19 cases from other non-COVID-19 events, such as pathogenic infections, stressors, alcohol consumption or other activities. Failure to detect COVID-19 can also occur owing to missing data. Participants did not wear the watches all the time, especially during sleep, when watches are often charged (especially in the case of the Apple Watch, which requires a longer charging time). We expect that this work will provide useful insights into improvement of smartwatches for health tracking.

Notably, classical methods for illness detection have generally relied on resting oral or skin temperature and comparison of an individual to a population average instead of their individual baseline. Many COVID-19 infections do not appear to cause a fever. Moreover, skin temperature is often measured using inaccurate devices, such as infrared devices, which are influenced by external temperatures and, thus, might not be the optimal method for infection detection. RHR and other longitudinal physiological measures might be valuable in conjunction with temperature measurements for early and specific disease detection. We previously found that Lyme disease can be detected pre-symptomatically using a smartwatch and pulse oximeter^6^, and here we show that *Mycoplasma* infection can be also identified (albeit not pre-symptomatically in this particular case). With continued development, wearable platforms, including those that use a variety of physiological parameters, can be used as a general method to monitor infectious diseases, chronic inflammation-related flares and other health-related signals to improve healthcare at both the personal and population levels. Consequently, the measurements described and data generated can enable global monitoring for future pandemic outbreaks.

## Methods

### Study participation

In total, 3,318 adult individuals 18–80 years of age were recruited for this study under protocol number 57022, approved by the Stanford University IRB. Participants were invited by social media, news and outreach to participants in previous studies. Participants registered using the REDCap survey system and were then asked to install the study app, called MyPHD (available for iOS and Android devices). The app transfers their wearable data (Fitbit via Fitbit secure OAuth 2.0 API; Apple Watch and Garmin via HealthKit repository and other HealthKit and Google Fit-compatible devices) to a cloud platform for the Stanford research team to perform analysis. Next, the NightSignal algorithm generated alerts (green and red) in real time, which were sent to the participants. Upon receiving alerts on the app, participants were asked to annotate events with surveys covering symptoms, activities, diagnoses, medications and vaccination. Alerts are visualized via a calendar, and participants annotate the alert for each day using the surveys described (Fig. 2, top).

In general, we expect that alerts can lead to reporting bias because participants who do not get alerts might not annotate events. However, we hope that visualizing alerts using a calendar (instead of notification-based alerts) encourages participants to annotate any day regardless of the alert color (green/normal or red/abnormal alerts). Indeed, some participants annotated their alerts daily. All participants who had at least 7 d of wearable data (NightSignal) received alerts (2,117 in total). The number of individuals who received alerts (2,117) was less than the number who enrolled (3,318). This might be due to participants who did not install the study app or had difficulties using it (for example, perhaps due to issues of language barriers) or had fewer than 7 d of data.

To evaluate the performance of the following algorithms, for COVID-19-positive cases, because we cannot precisely define the correct or incorrect alerts for each individual due to the unknown virus exposure time, we define an infection detection window as 21 d before the symptom onset for symptomatic cases or diagnosis date for asymptomatic cases and, hence, calculate the TPs and FNs at individual level based on the above infection detection window instead of an alert-based analysis.

### NightSignal

A previous study that investigated whether personal sensor data can help with COVID-19 detection showed that the daily RHR on its own does not allow substantial discrimination between participants who tested positive for COVID-19 and participants who tested negative for COVID-19; however, it has been shown that sleep and activity data have a considerable difference between the two groups^8^. We observed substantial performance improvement when overnight RHR approach is used compared to daily RHR, because overnight RHR avoids short-range non-infection events such as stress or intense exercise during the day (Extended Data Fig. 7); thus, we focused on nighttime signals.

#### Data pre-processing

**Extended Data Fig. 4:**
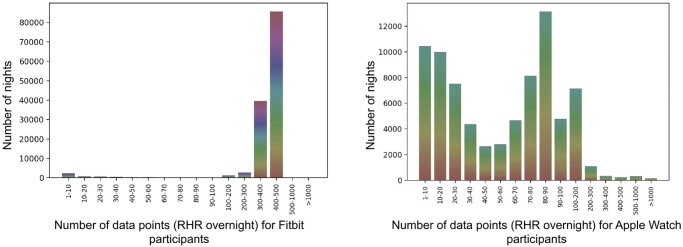
Comparing distribution of RHR overnight data in Fitbit vs. Apple Watch. To show the distribution of RHR data in Fitbit and Apple Watch, RHR overnight data points have been divided into 16 ranges (1-10, 10-20, etc.) and each bar depicts the total number of nights that falls into each group. Each participant is presented with a color. Note that for the majority of participants, for most of the nights, there are 300 to 500 RHR overnight data points (that is, almost minute resolution) in the case of Fitbit. However, the range differs considerably in Apple Watch; the reason is that Apple Watch takes heart rate and step counts readings with different resolutions based on the activities. Note that in the NightSignal algorithm, for each night, we use the RHR overnight data regardless of the amount of data. The reasons for not setting a threshold for the minimum amount of data points required are as follows: the first reason is that in most cases, even a very few data points are sufficient to get a proper average RHR overnight because we only consider HR records where the corresponding time interval (for example, few minutes) for step count is zero. The second reason for that is if we do so (for example, set the threshold to 40 data points), we will miss a significant number of nights (for example, first four bars). It is important to note that there were 15 participants who had data collected from both Fitbit and Apple Watch. With respect to the average RHR overnight that is used in NightSignal algorithm, except for one participant who had significant different values between the Fitbit and Apple Watch (one possible reason can be that the watch has been used by another person), for other 14 participants, the median delta between the Fitbit and Apple Watch data for joint nights (total of 1,006 nights) is only two bpm. Hence, despite the difference between the distribution of RHR overnight data points, there is no significant difference in the average RHR overnight point of view that is being used in the NightSignal algorithm between two devices. It is noteworthy to mention that unfortunately we did not have many COVID-19 positive cases with Garmin data to properly evaluate the algorithm for Garmin watches, and thus we did not include Garmin in our analysis.

**Extended Data Fig. 5:**
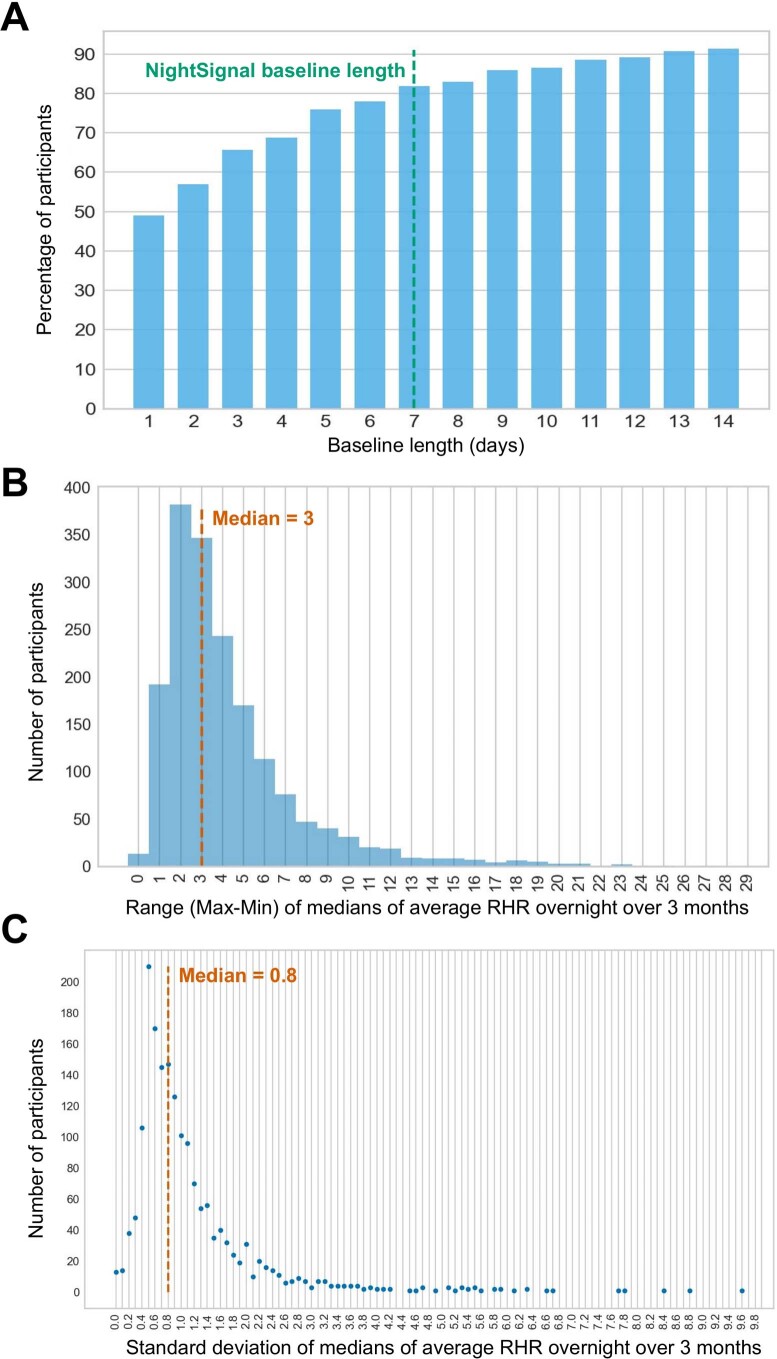
Thresholds and parameters in the NightSignal algorithm. As we discussed previously, the NightSignal algorithm uses the streaming median of average RHR overnight as the baseline. We believe that it is a proper individual healthy baseline as we show that the fluctuation is insignificant and it usually deviates due to a long-term abnormal event (for example, infection, medication consumption, vaccination). (**A**) The minimum number of nights required to hit a baseline close to the baseline over three months (within ± two bpm from the baseline - the reason behind choosing the threshold of two is that the baseline would still remain in the green zone) for the majority of participants. As depicted in the figure, for over 80% of the participants, the proper baseline was observed after only seven nights. (**B**) The range (max-min) of the medians of average RHR overnight during three months of data with the median value of only three bpm. Similarly, Quer et al. ^24^ studied the variability in individual resting heart rate and showed that most participants had a median weekly fluctuation in RHR of only three bpm. Given the fact that we only consider RHR overnight, the median fluctuation in RHR overnight is still three bpm even for a duration of three months. (**C**) The corresponding distribution of the medians of average RHR overnight over three months indicates a standard deviation that is very low (0.8).

**Extended Data Fig. 6:**
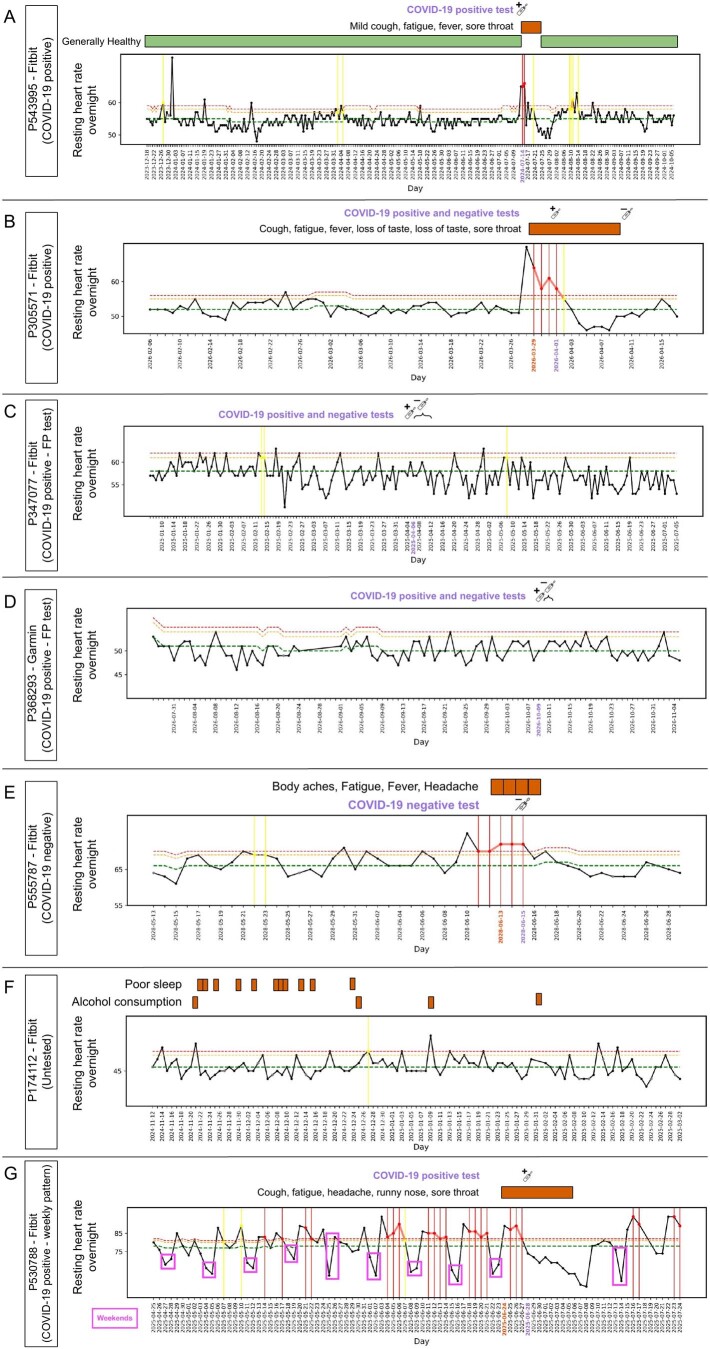
Additional examples of alerts for COVID-19 positive, COVID-19 negative, and untested participants. (**a**) Signals for a COVID-19 positive case with mild and short-lasting symptoms. Note that this participant received zero red alerts during a healthy period of over seven months and only received two red alerts starting the night of the symptom onset date (the only period where the RHR overnight is much higher than baseline for three consecutive nights). This example shows missing only two days of data can lead to missing a whole cluster of COVID-19 related alerts. (**b**) A COVID-19 positive case shows that triggering alerts based on deviations in two nights does not lead to delayed alert. (**c**) and (**d**) Both cases show participants who reported confirmed diagnosis of COVID-19 but with consecutive negative tests after the positive test, seven and two negative tests, respectively. In both cases, the NightSignal algorithm correctly did not trigger any red alert. (**e**) An example of a sick participant with moderate symptoms followed by a COVID-19 negative test. Note that RHR overnight period began to increase three nights before the symptoms developed. (**f**) An example of an untested participant who reported no illness or symptoms of any kind during the study (only some poor sleep and alcohol consumption points for P174112). This participant received no red alerts for over a three months healthy period, however alcohol consumption affects the RHR overnight but the impact is either only for one night or not severe enough to trigger a red alert. (**g**) This example indicates the impact of lifestyle changes (here weekdays vs. weekends) on RHR overnight in a COVID-19 positive participant. Clearly, repeated patterns occur before COVID-19 positive test and reoccurrence of the similar patterns begin two weeks after that (likely due to recovery from the illness and starting the same lifestyle).

**Extended Data Fig. 7:**
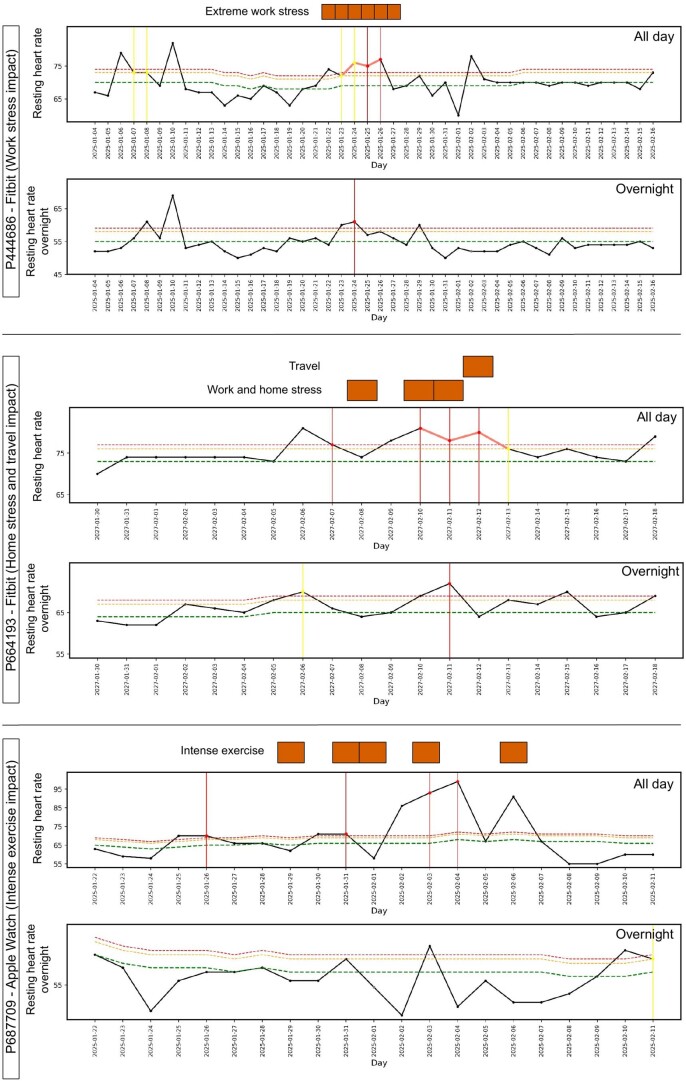
Impact of other non-infectious events on NightSignal alerts (all day vs. overnight). Examples of the impact of different events (for example, home and work stress, travel, and intense exercise) on the alerts based on two configurations: all day vs. overnight. Comparing the plots for each participant shows that the NightSignal algorithm reduces possible false positives due to non-infectious events by analyzing RHR overnight.

**Extended Data Fig. 8:**
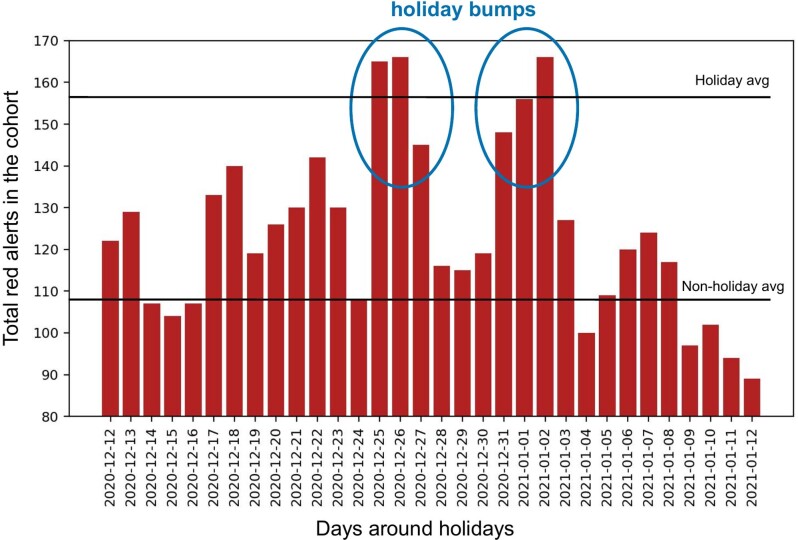
Impact of winter holidays on NightSignal alerts. As shown previously, there is a noticeable increase in the number of alerts during winter holidays — particularly late December and beginning of January — (‘holiday bump’) due to the higher rate of travel, alcohol, entertainment, stress, and illness compared to other times of the year.

The pre-processing stage provides consistency between different sources (that is, Fitbit and Apple Watch) and handles missing data. The resolution of the retrieved distinct raw heart rates and steps data from Fitbit and Apple Watch differs (Extended Data Fig. 4). To calculate the RHR overnight for different devices, first we consider the heart rate records where steps are zero and then aggregate the RHR values by calculating the average RHR during nighttime (that is, 24:00 to 7:00). In Extended Data Fig. 5a, we show that, for most participants (over 80%), median of average RHR overnight is a stable and reliable baseline, because, only after seven nights, it hits a baseline close to the baseline over 3 months. In the case of missing nights, we impute the values for only up to one night by calculating the average RHRs from the night before and immediately after the missed night.

#### Real-time alerting

**Extended Data Fig. 9:**
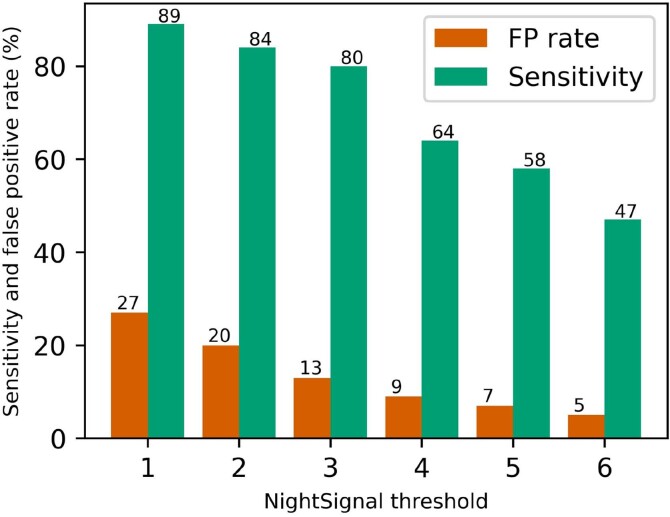
Sensitivity and false positive rate changes in the NightSignal algorithm for different thresholds. Under different thresholds, we report the sensitivity and false positive rate for the NightSignal algorithm. For the sake of clarity, it is important to mention that for anomaly detection problems like the one addressed in this study, having a perfect ground truth is very hard (here, due to the facts that the exact time of virus exposure is unknown in majority of the cases and also other non-infectious events can trigger the alerts - usually smaller clusters). Hence, defining the exact COVID-19 illness period and consequently correct alert is difficult. In other words, all the alerts within the assumed detection window might not necessarily be related to COVID-19, so a higher sensitivity does not by itself mean a greater accurate detection rate. Therefore, besides the aforementioned reasons in Supplementary Fig. 5, to keep the false positive rate sufficiently low, as well as achieving a high sensitivity, we use the threshold of three for the warning (yellow) level. Moreover, this threshold can be easily configured by each individual for a more personalized and precise analysis. This modification will require IRB approval and is outside of the scope of the present study and is thus part of a future work.

The NightSignal algorithm triggers the alerts based on the FSM shown in Extended Data Fig. 3. An FSM is defined by a list of its states, its initial state and the inputs (symbols) that trigger each transition and can produce an output based on a given input and/or a state. The NightSignal state machine, as depicted in Extended Data Fig. 3, contains six states and three outputs/colors (S_0_, S_1_ and S_2_ labeled with green alert, S_3_ and S_4_ labeled with yellow alert and S_5_ labeled with red alert) and three symbols as follows: (a) A_*i*_ < M_*i*_ + 3: for night *i*, the average RHR overnight is fewer than 3 beats per minute (b.p.m.) above the baseline (median of averages of RHR overnight for all nights up to night *i*). To keep the FP rate sufficiently low to avoid alarm fatigue, as well as achieving a high sensitivity (Extended Data Fig. 9), the threshold of 3 was chosen, because, for all participants, the median of fluctuation of medians of average RHR overnight over 3 months was only 3 b.p.m. (Extended Data Fig. 5b,c); (b) A_*i*_ = M_*i*_ + 3: for night *i*, the average RHR overnight is equal to 3 b.p.m. above the baseline; and (c) A_*i*_ > =M_*i*_ + 4: for night *i*, the average RHR overnight is greater than or equal to 4 b.p.m. above the baseline. Transition starts from initial state S_0_ (green alert), and the transition function takes one of the above six states and one of the above three symbols and returns a state with its corresponding label (alert).

### RHRAD

The current version of the AnomalyDetect online model is built based on the previous offline model from our previous study^7^. It uses RHR data and splits it into training data by taking the first 744 h as a baseline (1 month) and test data by taking the next 1-h data and uses a 1-h sliding window to find anomalies in the test data in ‘real time’ with a 0.02 threshold. If the anomalies occur frequently within 24 h, it will automatically generate either warning (yellow) or serious (red) alerts every 24 h. Red alerts were set if the anomalies occurred continuously for more than 5 h within each 24-h period; yellow alerts were set if the anomalies occurred for 1 h or continuously for fewer than 5 h; and green alerts were set if there were no anomalies.

### CuSum

We extended the CuSum online detection algorithm proposed in our previous work^7^ into the context of the real-time alerting system. Our previous work focused on the initial alarm for the purpose of early detection. For the setting of the alarm event, the trend of CuSum statistics was tracked using a 1-h resolution; the status of CuSum was evaluated every 12 h; and the alarm was reported each day.

As calculated in Mishra et al.^7^, the baseline was constructed from a 28-d sliding window in a personalized manner. If the data were missing for more than 14 successive days, the CuSum alerting system was restarted. The alerting system proceeded through chunks of data (56 d), and then the results from these different chunks were combined. Under the threshold of 95% quantile of CuSum statistics during the baseline, the difference of the standardized RHR residuals from the threshold was accumulated in each hour, and the zero-truncated positive difference was added to the stream of the CuSum statistics. When the CuSum statistics became significant for the first time compared to the statistics during the baseline (which serve as the null distribution), the initial alarm was triggered. After the initial alarm, the average CuSum statistics from each 12-h window were calculated, and the CuSum changed to a yellow status. If the CuSum statistics kept increasing in two successive 12-h intervals, a red status was recorded in the last 12-h period. If the CuSum statistics continued decreasing in two successive 12-h intervals, the status was turned back to green. In the case of all missing values in the 12-h intervals, the status was recorded as NA. The alarms were sent at 21:00 each day based on the latest recorded status.

### Isolation Forest

Isolation Forest is an unsupervised anomaly-detection model based on decision trees. We used the ensemble.IsolationForest class from the scikit-learn package in Python^23^ to isolate the observations by processing the randomly sub-sampled data in a tree structure and return the anomaly score of each sample and find the extreme points as anomalies. Isolation Forest uses a key parameter as contamination that sets the percentage of points in the data to be anomalous. A higher contamination level is more likely to generate more anomalies (that is, potentially higher FPs). In Supplementary Table 2, we report the results for two contamination-level settings: ‘auto’ and ‘0.095’. The value of 0.095 was chosen to have the same percentage of anomalies as in the NightSignal algorithm (that is, average of two alerts during a 21-d window for all COVID-19 positives, COVID-19 negatives and untested groups). Pre-processing and other parameters, such as resampling and overnight window, are the same as in the NightSignal algorithm.

In Fig. 3c, the cumulative scores of alerts are calculated by the formula (1) below. Let *D* = *{d*_*1*_*, d*_*2*_*, …, d*_*n*_*}* be a set of days and *R* = *{d*_*k*_*, d*_*k+1*_*, …, d*_*K+m*_*}* be a set of days where consecutive *k* alerts have occurred, and then the associated alert score for each day in the set *R* is calibrated to the size of the set *R* (that is, m + 1). Note that the most clustered alerts appear around the symptom onset date.

Formula (1):

$$\frac{{D\left( {\mathrm{days}} \right) = \left\{ {d_1,\,d_2, \ldots ,\,d_n} \right\},R\left ( {\mathrm {consecutive}}\,{\mathrm {alerts}}\right) = \left\{ {d_k,d_{k + 1}, \ldots d_{K + m}} \right\}}}{{S\left({\mathrm{alerts}}\,{\mathrm {scores}} \right) = \left\{ {s\left( {d_k} \right) = m + 1,\,s\left( {d_{k + 1}} \right) = m + 1, \ldots \,s\left( {d_{K + m}} \right) = m + 1} \right\}}}$$

### Visualization

Algorithm results were visualized using the matplotlib package, version 3.1.0.

### Reporting Summary

Further information on research design is available in the Nature Research Reporting Summary linked to this article.

## Online content

Any methods, additional references, Nature Research reporting summaries, source data, extended data, supplementary information, acknowledgements, peer review information; details of author contributions and competing interests; and statements of data and code availability are available at 10.1038/s41591-021-01593-2.

## Supplementary information

Supplementary Information Supplementary Tables 1–4

Reporting Summary

## Data Availability

De-identified raw heart rate and steps data used in this study can be downloaded at the following publicly available link: https://storage.googleapis.com/gbsc-gcp-project-ipop_public/COVID-19-Phase2/COVID-19-Phase2-Wearables.zip. Source data are provided with this paper.

## Source data

Source Data Fig. 4 COVID-19-positives source data and metadata

## Code availability

Code for the algorithms used in this study are publicly available at:

NightSignal algorithm: https://github.com/StanfordBioinformatics/wearable-infection

RHRAD algorithm: https://github.com/gireeshkbogu/AnomalyDetect/blob/master/scripts/rhrad_online_24hr_alerts_v6.py

CuSum algorithm: https://github.com/mwgrassgreen/Alarm

Isolation Forest algorithm: https://github.com/StanfordBioinformatics/wearable-infection/tree/main/isolationforest
