# Supplementary material for: Real-time alerting system for COVID-19 and other stress events using wearable data
Source: Nat Med. 2021 Nov 29;28(1):175–84. doi: 10.1038/s41591-021-01593-2 (PMC8799466; doi:10.1038/s41591-021-01593-2)
Supplement: Supplementary file 1 — Supplementary Tables 1–4 [file 41591_2021_1593_MOESM1_ESM.pdf]

nature portfolio

https://doi.org/10.1038/s41591-021-01593-2

Supplementary information

Real-time alerting system for COVID-19 and other stress events using wearable data

In the format provided by the authors and unedited

Table S1: Study Cohort

Number of participants: 3,318		COVID-19 positive	COVID-19 negative	Positives detected by NightSignal
**Age**				
Age range (Median)	19 - 79 (44)	19 - 70 (40)	19 - 79 (41)	19 - 70 (42)
**Sex**				
Female	1,854	160	801	46
**Ethnicity**				
Caucasian, European, White	2,631	213	1,008	55
Asian	151	10	65	1
Hispanic	120	24	45	5
Black or African-American	98	23	40	4
Mixed/Others/Undeclared	353	8	55	2
**Baseline medical history**				
None	1,217	99	483	25
Allergy or immune system disease or conditions	697	55	325	11
High blood pressure	505	35	218	10
High cholesterol	413	36	175	11
Respiratory or lung disease or conditions	321	27	153	8
**Baseline body mass index (BMI)**				
<25	1,237	92	557	20
25 to 30	1,373	84	368	22
>30	343	77	361	16
**Wearable data and alerts**				
Participants with wearable data	2,155			
Participants received real-time alerts	2,117			
Participants filled in at least one survey	2,122			
**COVID-19 positive and negative**				
With COVID-19 positive test	278			
With sufficient Fitbit data	49			
Prospective cases	19			
Retrospective cases	30			
With sufficient Apple Watch data	35			
Prospective cases	15			
Retrospective cases	20			
With COVID-19 negative test	1,213			
**COVID-19 vaccination**				
Received the first dose of COVID-19 vaccine	189			
Pfizer-BioNTech	99			
Moderna	90			
Received the first and second doses of COVID-19 vaccine	182			
Pfizer-BioNTech	94			
Moderna	88			

Table S2: Comparison of Performance of NightSignal, RHRAD, CuSum, and Isolation Forest Algorithms

Sensitivity (%)

	**NightSignal**	**NightSignal Fitbit Only**	**RHRAD**	**CuSum**	**IsolationForest** (contamination level = auto)	**IsolationForest** (contamination level = 0.095)
On COVID-19 positive population - during the infection detection window	67/84 = **80%**	38/49 = **77%**	29/42 = **69%**	29/40 = **72%**	64/84 = **76%**	32/84 = **38%**

Specificity (%)

		**NightSignal**	**NightSignal Fitbit Only**	**RHRAD**	**CuSum**	**IsolationForest** (contamination level = auto)	**IsolationForest** (contamination level = 0.095)
Alerts-based	On COVID-19 positive population - before the infection window	5,137/(5,137+713) = **87.8%**	3,553/(3,553+461) = **88.5%**	4,152/(4,152+585) = **87.6%**	3,530/(3,530+767) = **82.1%**	3,782/(3,782+902) = **80.7%**	4,286/(4,286+398) = **91.5%**
	On COVID-19 negative population - within 21 d of the test	10,241/(10,241+1,693) **= 85.8%**	5,886/(5,886+939) = **86.2%**	3,939/(3,939+577) = **87.2%**	3,613/(3,613+776) = **82.3%**	11,395/(11,395+3,510) = **76.4%**	13,094/(13,094+1,591) = **89.1%**
	On untested population - the whole time frame	76,883/(76,883+10,493) = **87.9%**	51,222/(51,222+7,032) = **87.9%**	32,816/(32,816+4,643) = **87.6%**	31,838/(31,838+6,113) = **83.8%**	88,262/(88,262+23,908) = **78.6%**	100,649/(100,649+10,903) = **90.2%**
Individuals-based	On COVID-19 positive population - before the infection window (avg)	**88.6%**	**88.8%**	**88.0%**	**82.3%**	**79.2%**	**90.9%**
	On COVID-19 negative population (avg) - within 21 d of the test	**86.4%**	**86.4%**	**87.3%**	**82.7%**	**76.4%**	**89.0%**
	On untested population (avg) - the whole time frame	**88.4%**	**88.0%**	**87.5%**	**84.2%**	**77.2%**	**90.1%**

Table S3: Comparison of Alerts for COVID-19 and Non-COVID-19 Participants

Participant category		Mean no. of red alert days (NightSignal) per person during a 21 day window
COVID-19 positive population (infection detection period)	Symptomatic	3.36
	Asymptomatic	3.64
	Both	3.42
COVID-19 negative population (pre-test period)		1.30
Untested population (any period)		1.09
All non COVID-19 positive population (any period)		1.15

Table S4: Participants' Experience with Alerts

Survey for participants who received alerts	Number of reports	Examples of feedback comments (7 examples for each category)
The number of red alerts is acceptable to me	**235 (73%)**	- Most of the times when I have had a red alert, I was not feeling well, or I was traveling and my schedule was off<br>- Told me when I was sick.<br>- They were on days I was not feeling well.<br>- The alerts do detect events.<br>- It was because I got the second vaccine. It might have picked up the mono diagnosis.<br>- The days of red alerts actually did correlate to the days I was not feeling well.<br>- I have had 2 red alerts, and they both reflected very stressful situations I encountered. So to me, that seems impressive and right on target!
I am getting less red alerts than expected	**67 (21%)**	- There are days I'm very tired, slept poorly, drank alcohol etc and I would expect those days to be red and they aren't.<br>- On days I forgot to put on my Fitbit, there were practically no alerts noted. Also when flying, last year I received them and none this year.<br>- Based on nighttime heart rate alone I would have expected it to flag at least a couple of instances - there is a general pattern that I have when I haven't slept well and feel tired - regardless of what the Fitbit app or MyPHD indicates.<br>- Barely had any red alerts on my time with the study.<br>- I had a recent period of daily shortness of breath and high fatigue, with no alerts. I know it hasn't been Covid, but I have still been surprised it hasn't shown up in the last month.<br>- Some days I feel under the weather and get no alerts.<br>- There were definitely days where my activity was abnormal, but the alerts were all green.
I am getting many false red alerts, and I am feeling harassed by the high frequency of red alerts	**18 (6%)**	- I'm not getting harassed, but I can not correlate any activities with the red days.<br>- I appreciate them, but am frequently uncertain as to why I get a red alert. More information would be appreciated. Also, I note, that they appear to be more linked to my cycle than illness.<br>- Many days i was physically active and feeling fine i was getting a red alert. Fitbit was rarely syncing with the program (i am constantly having fitbit problems).<br>- I wouldn't say it was "many" red alerts, but definitely mostly false positives. I got several red alerts, but was only actually sick once (questionable if I was actually sick). It definitely wasn't Covid<br>- The alerts do not seem applicable.<br>- I have a number of red alerts on days when I feel fine and have no idea what might be different. There are often several days in a row like this, once per month, which makes me wonder if it might be linked to my cycle.<br>- Harassed is an overstatement, but there are many false red alerts.
