# Supplementary material for: Real-time alerting system for COVID-19 and other stress events using wearable data
Source: Nat Med. 2021 Nov 29;28(1):175–84. doi: 10.1038/s41591-021-01593-2 (PMC8799466; doi:10.1038/s41591-021-01593-2)
Supplement: Supplementary file 2 — Reporting Summary [file 41591_2021_1593_MOESM2_ESM.pdf]

nature research

Corresponding author(s): Amir Bahmani
Michael Snyder

Last updated by author(s): Oct 21, 2021

Reporting Summary

Nature Research wishes to improve the reproducibility of the work that we publish. This form provides structure for consistency and transparency in reporting. For further information on Nature Research policies, see our Editorial Policies and the Editorial Policy Checklist.

Statistics

For all statistical analyses, confirm that the following items are present in the figure legend, table legend, main text, or Methods section.

n/a	Confirmed	
☐	☒	The exact sample size (*n*) for each experimental group/condition, given as a discrete number and unit of measurement
☐	☒	A statement on whether measurements were taken from distinct samples or whether the same sample was measured repeatedly
☒	☐	The statistical test(s) used AND whether they are one- or two-sided<br>*Only common tests should be described solely by name; describe more complex techniques in the Methods section.*
☐	☒	A description of all covariates tested
☐	☒	A description of any assumptions or corrections, such as tests of normality and adjustment for multiple comparisons
☐	☒	A full description of the statistical parameters including central tendency (e.g. means) or other basic estimates (e.g. regression coefficient) AND variation (e.g. standard deviation) or associated estimates of uncertainty (e.g. confidence intervals)
☒	☐	For null hypothesis testing, the test statistic (e.g. F, t, r) with confidence intervals, effect sizes, degrees of freedom and P value noted<br>*Give P values as exact values whenever suitable.*
☒	☐	For Bayesian analysis, information on the choice of priors and Markov chain Monte Carlo settings
☒	☐	For hierarchical and complex designs, identification of the appropriate level for tests and full reporting of outcomes
☒	☐	Estimates of effect sizes (e.g. Cohen's *d*, Pearson's *r*), indicating how they were calculated

Our web collection on statistics for biologists contains articles on many of the points above.

Software and code

Policy information about availability of computer code

Data collection	MyPHD Mobile development: Swift 4 and Java - Xcode (12.4) and Android Studio (4.1.2)<br>Cloud Computing services: GCP BigQuery (1.22.0), GCP Storage (1.22.0), GCP Cloud Functions, Terraform (0.12.29)<br>REDCap (11.2.4)
Data analysis	All statistical analyses were performed in python 3.6 and R 3.3.0 and the Python sklearn version 0.23.1. and R xts packages version 0.12.1.<br>Results were visualized using the matplotlib package version 3.1.0.<br>NightSignal algorithm: https://github.com/StanfordBioinformatics/wearable-infection<br>RHRAD algorithm: https://github.com/gireeshkbogu/AnomalyDetect/blob/master/scripts/rhrad_online_24hr_alerts_v6.py<br>CuSum algorithm: https://github.com/mwgrassgreen/Alarm<br>Isolation Forest algorithm: https://github.com/StanfordBioinformatics/wearable-infection/tree/main/isolationforest

For manuscripts utilizing custom algorithms or software that are central to the research but not yet described in published literature, software must be made available to editors and reviewers. We strongly encourage code deposition in a community repository (e.g. GitHub). See the Nature Research guidelines for submitting code & software for further information.

Data

Policy information about availability of data

All manuscripts must include a data availability statement. This statement should provide the following information, where applicable:

- Accession codes, unique identifiers, or web links for publicly available datasets
- A list of figures that have associated raw data
- A description of any restrictions on data availability

Data availability:

Source Data are available with this paper. De-identified raw heart rate and steps data used in this study can be downloaded from the following publicly available link: https://storage.googleapis.com/gbsc-gcp-project-ipop_public/COVID-19-Phase2/COVID-19-Phase2-Wearables.zip

Field-specific reporting

Please select the one below that is the best fit for your research. If you are not sure, read the appropriate sections before making your selection.

☒ Life sciences ☐ Behavioural & social sciences ☐ Ecological, evolutionary & environmental sciences

For a reference copy of the document with all sections, see nature.com/documents/nr-reporting-summary-flat.pdf

Life sciences study design

All studies must disclose on these points even when the disclosure is negative.

Sample size	The most crucial aspect of the study was to compare the participant's continuous time-series data to their own baseline to detect COVID-19 infection in real-time and return the results in the form of alerts to participants via the study app, MyPHD. Thus, in terms of sample size, the crucial aspect was to ensure maximal sampling within an individual before and around the time of COVID-19 infection (i.e. symptom onset for symptomatic cases and diagnosis date for asymptomatic cases). For analysis, we used 84 COVID-19 positives (45 confirmed via written documentation or verbal confirmation) who had (1) COVID-19 diagnoses date, (2) were able to provide symptom onset and/or diagnosis dates, and (3) had data recorded from wearables spanning and adjacent to the dates of the COVID-19 infection. We recruited as broadly as possible, and tried to maximize the number of individuals who fit these criteria, but were limited by the infection rates during the recruitment period of the study. Out of the 3,318 individuals enrolled, and 2,155 who were wearing fitness trackers, we identified 278 individuals with COVID-19 infection. Of these, 84 individuals were wearing the devices around the infection time; hence, this subset of individuals was chosen for analysis. We also added 1,213 participants who reported a COVID-19 negative test, 1,825 participants without any COVID-19 test report, and 189 participants who received the COVID-19 vaccine (Moderna or Pfizer-BioNTech), among them, 182 participants were fully vaccinated (i.e. both doses).
Data exclusions	All available data were used for analyses. There were no data excluded from the analyses, except in cases where data from the wearbles was missing during or just prior to a self-reported COVID-19 infection.
Replication	This was an observational study in which we did not perform experiments.
Randomization	Participants were not randomized. There was no allocation to groups. We recruited individuals from 3 distinct groups, COVID-19 positive, COVID-19 negatives, and untested individuals (Vaccinated individuals could be either COVID-19 positive, negative, or untested) but the only group-based analysis was to compare alerts duration and intensity in each group. The main detection algorithms were run on each individual participant's data separately. Each participant's longitudinal data as used to construct participant-specific heart-rate baselines, deviations from the baseline, and corresponding alerts were analyzed in a participant-specific manner.
Blinding	Blinding was not relevant to the study (that is, there was no allocation to groups or interventions).

Reporting for specific materials, systems and methods

We require information from authors about some types of materials, experimental systems and methods used in many studies. Here, indicate whether each material, system or method listed is relevant to your study. If you are not sure if a list item applies to your research, read the appropriate section before selecting a response.

Materials & experimental systems

n/a	Involved in the study	
☒	☐	Antibodies
☒	☐	Eukaryotic cell lines
☒	☐	Palaeontology and archaeology
☒	☐	Animals and other organisms
☐	☒	Human research participants
☒	☐	Clinical data
☒	☐	Dual use research of concern

Methods

n/a	Involved in the study	
☒	☐	ChIP-seq
☒	☐	Flow cytometry
☒	☐	MRI-based neuroimaging

Human research participants

Policy information about studies involving human research participants

Population characteristics	The mean age of the 5,262 participants at time of enrollment was 44 (range, 19–79); 55.8% were women. However, out of the 278 COVID-19 positive individuals , 160 (57.5%) were women. The self-reported ethnic distribution of the full cohort was 79.3% European/Caucasian/White, 4.5% Asian, 3.6% Hispanic, 2.9% African American, 10.6% Mixed/Other/Undeclared. The most common self-reported health conditions at entry were allergy or immune system disease or conditions, high blood pressure, high cholesterol, and respiratory or lung disease or conditions.

Recruitment	Participants were recruited by social media, news, and outreach to participants in previous studies. Since social media was one of the recruitment methods used, there could be a bias towards people who use social media. The use of wearable devices likely biases our study cohort towards individuals of higher socioeconomic-strata, who are more likely to be able to afford the devices. It is also possible that individuals who own wearables devices are more interested in using wearable devices to monitor both activity and health. However, we do not think that the results of our algorithm are affected by interest in wearable devices especially as the NightSignal algorithm is not restricted to one type of wearable devices.
Ethics oversight	Stanford IRB (#57022) and Data Risk Assessment (#665)

Note that full information on the approval of the study protocol must also be provided in the manuscript.
